# Single Cycle Structure-Based Humanization of an Anti-Nerve Growth Factor Therapeutic Antibody

**DOI:** 10.1371/journal.pone.0032212

**Published:** 2012-03-05

**Authors:** Sonia Covaceuszach, Sara Marinelli, Ivet Krastanova, Gabriele Ugolini, Flaminia Pavone, Doriano Lamba, Antonino Cattaneo

**Affiliations:** 1 Lay Line Genomics, SpA, Roma, Italy; 2 Istituto di Cristallografia, Consiglio Nazionale delle Ricerche, Trieste, Italy; 3 Istituto di Biologia Cellulare e Neurobiologia, Consiglio Nazionale delle Ricerche, Roma, Italy; 4 Structural Biology Laboratory, Sincrotrone Trieste, S.C.p.A., Trieste, Italy; 5 European Brain Research Institute, Roma, Italy; 6 Scuola Normale Superiore, Pisa, Italy; National Institute for Medical Research, Medical Research Council, London, United Kingdom

## Abstract

Most forms of chronic pain are inadequately treated by present therapeutic options. Compelling evidence has accumulated, demonstrating that Nerve Growth Factor (NGF) is a key modulator of inflammatory and nociceptive responses, and is a promising target for the treatment of human pathologies linked to chronic and inflammatory pain. There is therefore a growing interest in the development of therapeutic molecules antagonising the NGF pathway and its nociceptor sensitization actions, among which function-blocking anti-NGF antibodies are particularly relevant candidates.

In this respect, the rat anti-NGF αD11 monoclonal antibody (mAb) is a potent antagonist, able to effectively antagonize rodent and human NGF in a variety of *in vitro* and *in vivo* systems. Here we show that mAb αD11 displays a significant analgesic effect in two different models of persistent pain in mice, with a remarkable long-lasting activity. In order to advance αD11 mAb towards its clinical application in man, anti-NGF αD11 mAb was humanized by applying a novel single cycle strategy based on the *a priori* experimental determination of the crystal and molecular structure of the parental Fragment antigen-binding (Fab). The humanized antibody (hum-αD11) was tested *in vitro* and *in vivo*, showing that the binding mode and the NGF neutralizing biological activities of the parental antibody are fully preserved, with even a significant affinity improvement. The results firmly establish hum-αD11 as a lead candidate for clinical applications in a therapeutic area with a severe unmet medical need. More generally, the single-cycle structure-based humanization method represents a considerable improvement over the standard humanization methods, which are intrinsically empirical and require several refinement cycles.

## Introduction

The neurotrophin Nerve Growth Factor (NGF) [1] exerts a wide range of physiological functions not only in the development and maintenance of specific neuronal populations of the vertebrate nervous system [2], [3], but also in some non neuronal cells, including cells of the immune system such as mast cells, basophils and monocytes [4]. It is worthy of note that, besides its broad range of physiological effects, NGF is involved in several disease states such as in certain chronic inflammatory or neuropathic pain states [5], [6] and in several human malignancies [7].

There has been an increasing recognition that NGF regulates the function of adult peripheral sensory neurons including small-diameter nociceptive afferents, thereby exerting a pain modulation activity through nociceptor sensitization [8]. Interestingly, NGF-induced activation of the Tropomyosin-related receptor kinase A (TrkA) receptor on mast cells as well as on macrophages and monocytes recruited at an injured or inflamed site determines the release of mediators that further contribute to the sensitization of sensory nociceptors [6]. Therefore, NGF modulates pain responses and changes pain thresholds by two principal mechanisms: a direct TrkA-mediated activation of pain signaling through receptors and channels on nerves such as Transient Receptor Potential cation channel subfamily V member 1 (TRPV1) and TetrodoToXin (TTX) insensitive voltage-gated sodium channel Na_v_, and indirectly through the TrkA mediated degranulation of mast cells and basophils.

Thus, the NGF-TrkA system appears to be a master control system for pain, in spreading inflammation and increasing the electrical neuronal response in nerve endings, functionally placed upstream in the hierarchy of the pain regulation process.

Besides a large body of evidence in animal models, the clinical relevance of the functional role of the NGF-TrkA system in pain has received considerable and compelling validation in humans. First of all, increased NGF levels are found in inflamed tissues and fluids from patients with pathological conditions such as arthritis [9], pancreatitis and prostatitis [10]. In humans, exogenous NGF infusions either locally or systemically, induce pain [11]. Finally, humans harboring mutations in the NGFB [12], [13] and TrkA genes [14] suffer congenitally from a complete loss of pain sensations, leading to severe self-mutilation.

For all these reasons, there has been a great interest in the development of antagonists of NGF as analgesic drugs for chronic and inflammatory pain conditions [15] such as osteoarthritis [16]. In this respect, antibodies against NGF constitute the strategy of choice to antagonize the actions of NGF, ever since the pioneering “immunosympathectomy” experiments by Levi-Montalcini [1], [17]. Indeed, the potent analgesic effects of anti-NGF antibodies have been well documented in a variety of animal pain models [6].

The rat anti-NGF monoclonal antibody (mAb) αD11 [18] deserves a special interest, as a therapeutic candidate, because it binds mouse NGF (mNGF) with picomolar affinity [19] with no cross-reactivity towards closely related members of the neurotrophin superfamily [20] and antagonizes very effectively its biological function in a variety of *in vitro* and *in vivo* systems [21], [22], [23], [24], [25].

In this study we demonstrate the potent and remarkably long lasting analgesic activity of the mAb αD11 on different rodent models of tonic/chronic pain. In order to pursue its therapeutic development, mAb αD11 was humanized by a novel strategy, exploiting the *a priori* 3D crystal structure determination of the parental rat Fab αD11 (PDB_ID: 1ZAN) [26], [27]. This resulted to be a crucial approach, that allowed to humanize αD11 antibody variable regions, by Complementary Determining Regions (CDRs) grafting, in a single cycle, obtaining a humanized version (hum-αD11) whose binding characteristics and NGF antagonizing activity, both *in vitro* and *in vivo*, are fully preserved with respect to the parental rat counterpart.

## Results

### Mab αD11 binds human NGF equally well as mouse NGF

**Figure 1:**
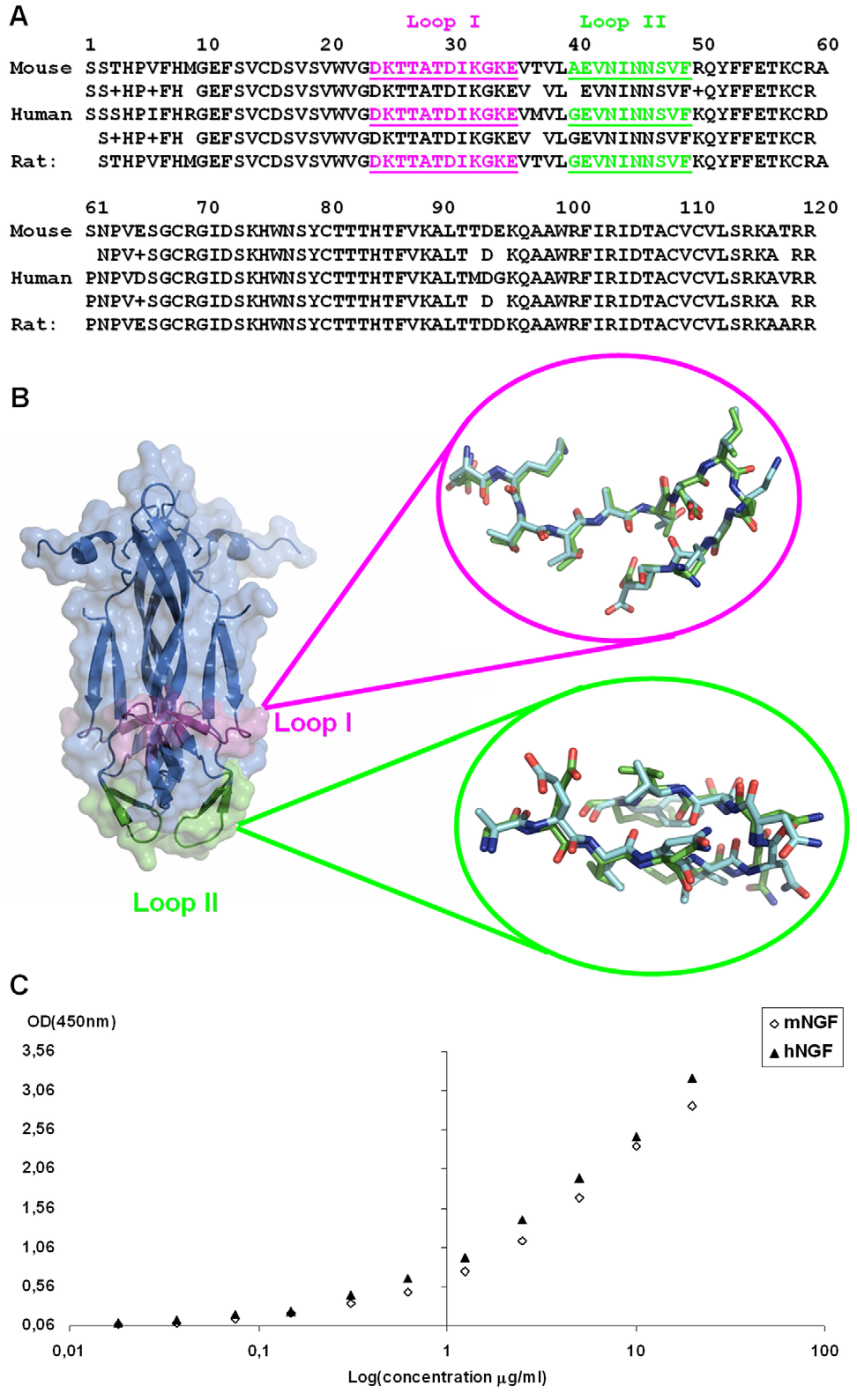
*In vitro* binding affinity of the parental mAb αD11 towards hNGF and mNGF. (a) Sequence alignment of mNGF, hNGF and rat NGF (Loop I and Loop II are colored in magenta and green, respectively). (b) Overall structure of NGF and structural comparisons [30], [31] of Loop I (in magenta) and Loop II (in green) from mNGF (PDB_ID: 1BTG, cyan) [28] and hNGF (PDB_ID: 1WWW, green) [29]. This Figure was prepared with PyMOL [32]. (c) ELISA assay with mNGF and hNGF coating (5 µg/ml) and serial dilutions of parental mAb αD11. The experiments were done in duplicate.

A basic requirement for mAb αD11 to be employed in human clinical applications is that its binding affinity for hNGF should be comparable to that for rodent NGF. The epitope recognized by mAb αD11 was identified in Loops I and II of mNGF. While the sequences of rat and human NGF in this region are identical, for the mouse and human NGF they differ at position 40 (**Figure 1A**). A structural alignment of Loop I and Loop II of the mouse and human NGF crystallographic structures (PDB_ID: 1BTG, PDB_ID: 1WWW) [28], [29] respectively, shows a good superposition (**Figure 1B**).

Thus, we can reliably predict that mAb αD11 binds to hNGF equally well as to mNGF. Indeed, an ELISA assay, with solid-phase coated mNGF and hNGF and serial dilutions of mAb αD11, confirms that mAb αD11 recognizes hNGF and mNGF with a comparable affinity (**Figure 1C**). At the functional level, the potency of mAb αD11 to neutralize the activity of NGF from different species was ascertained by the TF-1 cell proliferation assay [33] exhibiting a similar concentration-dependent inhibition of cell proliferation for human, rat and mouse NGF, respectively (data not shown).

### *In vivo* analgesic properties of anti-NGF mAb αD11 on formalin-induced pain and on neuropathic pain

The antagonistic properties of mAb αD11 are well established, as this anti-NGF antibody is extremely effective at neutralizing the biological actions of NGF in a wide variety of *in vivo* systems [20], [21], [22], [23], [24], [25], thanks to its extremely high binding affinity [19] and epitope specificity [20], [27]. In order to confirm the therapeutic potential of the αD11 antibody, its analgesic properties were assessed *in vivo* on two different models of tonic/chronic pain in mice.

**Figure 2:**
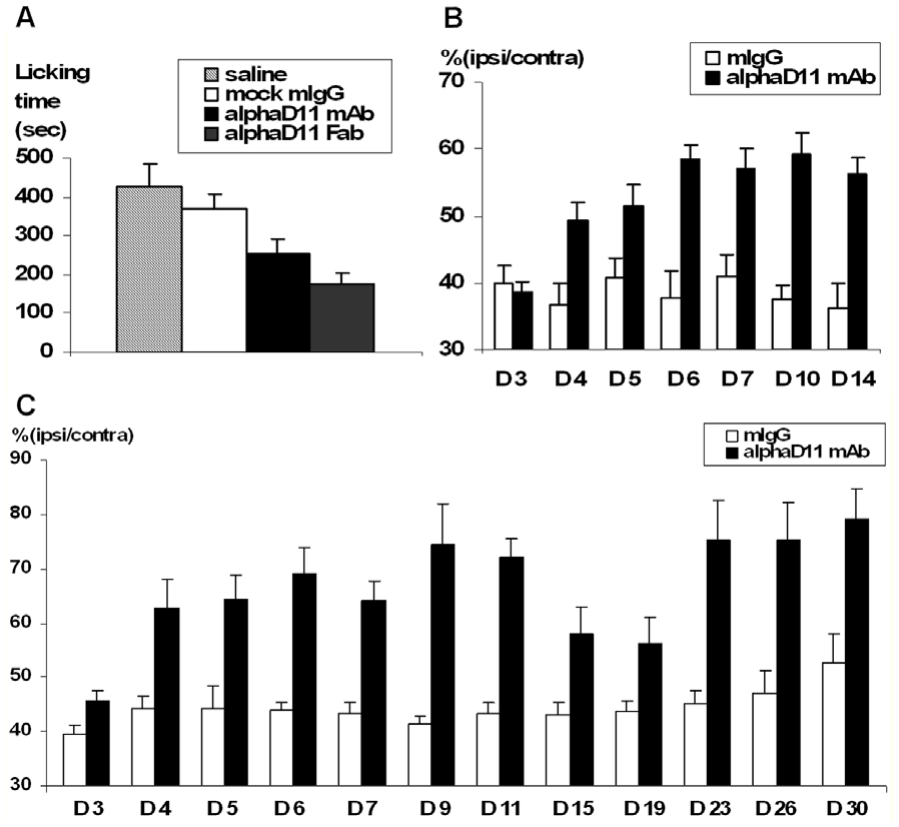
*In vivo* analgesic activity of parental mAb αD11 in inflammatory and neuropathic pain models. (a) Analgesic effects of αD11 antibody administration on the late phase (15–40 min) in the course of the formalin test. Treatment consisted in saline (negative control) or antibody injection (single doses: 12.5 µg of mock mouse mAb or two different molecular formats of αD11, *i.e.* mAb or Fab) performed (in the same paw as for formalin) 45 min before formalin injection and testing. Statistical analysis was performed on each phase (ANOVA and Fisher's Test for comparison of each couple of groups). Each experimental group included at least 8 animals. (b) Analgesic effects of αD11 antibody in the short lasting protocol of neuropathic pain model: mAb αD11 significantly increased the value of ipsilateral/contralateral index (ratio between the threshold forces measured for the two hind paws, the one ipsilateral to surgery and the contralateral one. Mean value ± s.e.), starting from day 4 to day 14, one week after the last antibody injection. Control mice were injected with either mock mouse IgG, (1.4 mg/Kg) or saline solution (sal). ANOVA test for repeated measures resulted in statistical significance for treatment (p<0.0001), time (p<0.0001) and the interaction between the two factors (treatment×time) (p<0.0001). (c) Analgesic efficacy of mAb αD11 (one dose: 2 mg/kg) in the long lasting protocol of neuropathic pain model. MAb αD11 increased the ipsilateral/contralateral index, starting either from day 5. The analgesic effect, which disappeared around days 17–19, increases again to reach a plateau between day 27 and day 31, identifying a late phase in the action of mAb αD11 (long-term effect). ANOVA test for repeated measures resulted in statistical significance for treatment (p<0.005), time (p<0.005) and the interaction between two factors (treatment×time) (p<0.005).

In the formalin-induced inflammatory pain model, formalin injection resulted in the typical biphasic response with the highest peak after 5 min and a second phase of licking that started 15 min after the treatment. The mAb αD11 was administered, either as IgG or Fab fragment format, 45 min before formalin injection and showed a significant analgesic effect (**Figure 2A**) clearly specific for the second phase (late inflammatory phase, *i.e.* 15–40 min) of the pain response. The analgesic effect was superior for the mAb αD11 in the Fab format, by halving the response of persistent pain, as compared either to saline (p<0.01) or to control mAb treatment (p>0.05) (**Figure 2A**). The strong analgesic potency of Fab αD11 in relation to that of the whole IgG counterpart, may be due to its higher diffusion rate and hence greater tissue penetration and bioavailability.

The analgesic potency of mAb αD11 was further evaluated in a mouse model of neuropathic pain, the Chronic Constriction Injury (CCI) of the sciatic nerve [34], following two treatment protocols, a short and a long lasting protocol (see Materials & Methods). In both protocols (**Figure 2B** and **Figure 2C**), mAb αD11 (Intra-Peritoneal injected (I.P.)) exhibited a very significant analgesic effect, as compared to mouse IgG mock. In a first set of experiments (short protocol) (**Figure 2B**), four I.P. injections of mAb αD11 (from day 3 to day 6 after ligation of the nerve) were able to significantly reduce mechanical allodynia, starting from day 4 after surgery. On this basis, a second set of experiments with a longer observation period (long lasting protocol, observation up to 31 days following sciatic nerve ligature), was performed. The observation of animals undergoing long lasting protocol revealed a quite unexpected temporal profile for the strong analgesic activity induced by mAb αD11 (**Figure 2C**). Two phases can be recognized in the analgesic action: the first one identifies a pharmacological effect of mAb αD11 (an effect which declined in parallel with the drop of the antibody concentration in circulating blood, reaching a minimum analgesic effect around day 17, *i.e.* one week after the end of the treatment). After the gradual decline of the anti-allodynic effect, in the second phase (from day 21 to day 31) mAb αD11 again reduced neuropathic pain, displaying a long-term analgesic effect of the anti-NGF antibody (**Figure 2C**). This long lasting analgesic effect is likely to involve persistent changes in gene expression in sensory neurons, demonstrating that αD11 antibody is not just as a potent analgesic, but also a long-term disease-modifying drug.

### Structure-based humanization design of anti-NGF mAb αD11

In order to advance its development towards clinical evaluation in patients, mAb αD11 was humanized by a novel structure-based strategy, taking advantage of the available 3D crystal structure of the rat Fab αD11 (PDB_ID: 1ZAN) [26], [27]. The structural information gained from the rat Fab αD11 crystallographic structure was exploited to optimize the selection of the acceptor human antibody framework regions (FWRs), onto which the CDRs of the donor murine anti-NGF mAb αD11 were grafted. The acceptor human FWRs for mAb αD11 humanization were selected by a novel approach [35], based on a comparison of both the primary (% sequence homology/identity) and the tertiary structures (degree of structural similarity based on the root mean square deviations (r.m.s.d) calculated by taking into account the C^α^ skeleton atoms) of the parental antibody with all the available experimental crystal structures of human and humanized antibodies. A cluster of candidate human FWRs acceptors was identified, scored on the basis of the highest level of homology/identity of the sequences present in the Protein Data Bank [36], for which high resolution X-ray structural data (*i.e.* not lower than 2.5 Å) were available. The search was performed at first by considering the overall antibody variable regions (*i.e.* the F_v_ fragment) and then by narrowing down to the FWRs. The selected crystallographic structures were superimposed to that of the parental F_v_αD11, using the “*superimpose*” software [37], and the r.m.s.d for each individual structural comparison were calculated, considering only the C^α^ atoms at the correspondent positions, on the respective backbones, closer than 2.0 Å. Therefore, the selection of the optimal human FWRs acceptors for humanization was configured as a three-variable problem, resulting in a 3D plot that combined the information from the primary structure comparison (% sequence homology/identity both at the level of the F_v_ or only of the FWRs) and from the tertiary structure alignment (the degree of structure similarity expressed in terms of r.m.s.d. and by the % of C^α^ atoms employed in the r.m.s.d. calculations).

**Figure 3:**
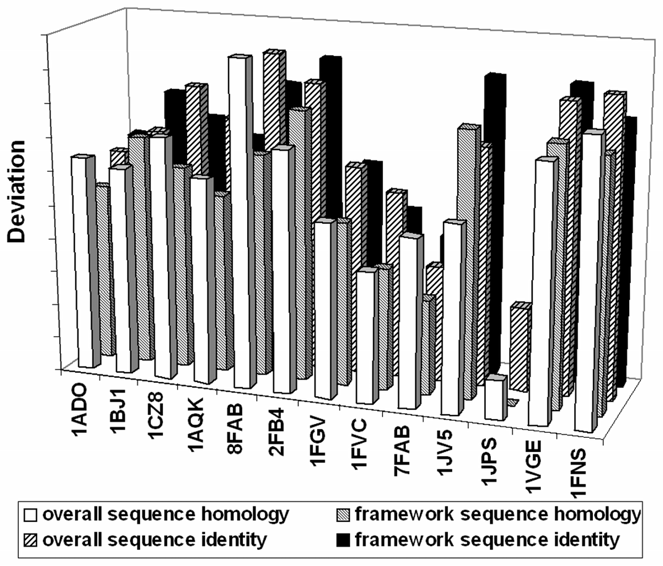
Selection of acceptor human FWRs for mAb αD11 humanization. Deviations of parental Fab αD11 from each of the selected human and humanized antibody, taking into account the sequence alignment and structural comparison (calculated considering both the overall sequence homology and identity percentage and the percentage of sequence homology and identity restricted to FWRs).

**Figure 4:**
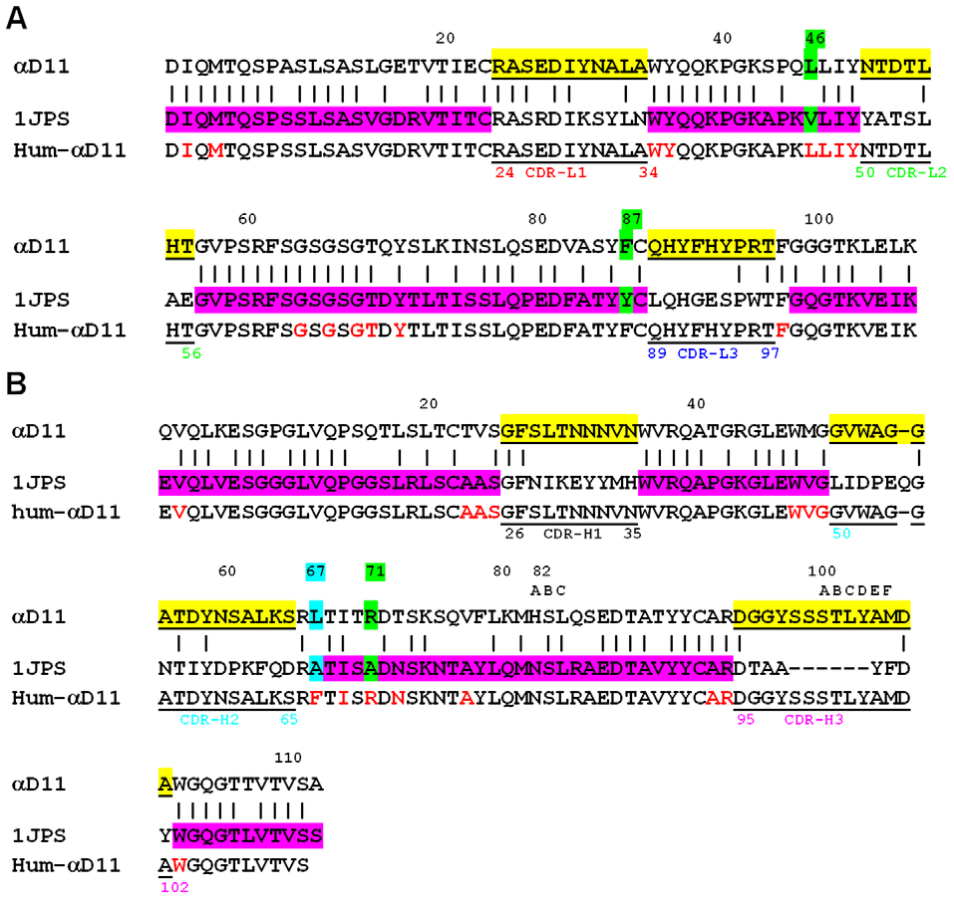
Primary structure comparison. Sequence alignment of V_κ_ (A) and V_H_ (B), respectively of parental Fab αD11 (PDB_ID: 1ZAN) [26], [27] with the selected template for humanization (PDB_ID: 1JPS) [38] and hum-αD11, obtained by CDRs grafting (highlighted in yellow) on PDB_ID: 1JPS [38] FWRs (highlighted in magenta) with the retro-mutations (highlighted in green) and the mutation (highlighted in cyan) . The six CDRs are underlined and the residues belonging to the Vernier zones are colored in red. The residues numbering is according to Kabat [39].

**Figure 5:**
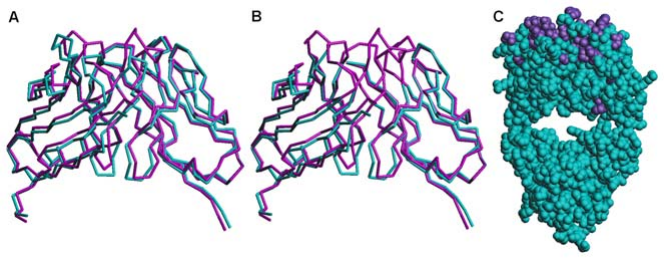
Tertiary structure comparison. Structural alignment between the variable domains of parental Fab αD11 (PDB_ID. 1ZAN) [26], [27] in magenta (a) with the selected FWRs acceptor 1JPS [38] (in cyan) and (b) with the model of the resulting humanized antibody after CDRs grafting (whose FWRs are depicted in cyan, while its CDRs are in magenta); (c) Model of the hum-αD11 after CDR grafting and mutagenesis in the chosen FWRs. The residues of human and animal origin are highlighted in cyan and violet, respectively.

As shown in **Figure S1**, the 3D distributions of the clusters were mutually consistent for all of the combinations of the variables taken into account. Moreover, by comparing the distances (**Figure 3**) between the point of each of the selected acceptors to the one having the coordinates corresponding to 100% sequence homology/identity both at the level of the variable regions of the heavy (V_H_) and of the light (V_κ_) chains F_v_ or only of the FWRs, 0.00 Å r.m.s.d. and 100% of C^α^ atoms employed in the r.m.s.d. calculations, *i.e.* the ideal human or humanized antibody, it was straightforward to unequivocally identify the best FRWs acceptor candidate among the human or humanized antibody of choice, both at the levels of the primary and tertiary structures. Thus, on the basis of the described method, the humanized antibody (PBD_ID: 1JPS) [38] was chosen as acceptor FWRs in the ensuing process of CDR grafting in the humanization of the αD11 antibody. The similarity of their FWRs is displayed at the level of their F_v_ region, both by sequence alignment (**Figure 4**) and the 3D structural superimposition (**Figure 5A**).

In order to design the final humanized form of the αD11 antibody (hum-αD11), first the CDRs residues of the parental mAb αD11 (underlined in **Figure 4**) were combined with the FWRs of PDB_ ID: 1JPS [38] (CDR grafting). A few retro-mutations were introduced, as follows: positions L46 and L87 were mutated (to the rat mAb αD11 residue at this position) to preserve the interface between V_H_ and V_κ_, whilst the H71 position was retro-mutated (A→R) to maintain the characteristics of the Vernier zones [39], [40]. Subsequently, following the comparison with the main consensus sequences of human immunoglobulins, a H67 (L→F) mutation was introduced, considering that the residue in this position was unusual both in the donor and in the acceptor FWRs, being therefore substituted by a well conserved residue in human antibodies. The resulting alignment of the Fv of hum-αD11 to the donor (parental αD11) and acceptor (PDB_ID: 1JPS) [38] sequences is presented in **Figure 4**.

The structural model of the designed hum-αD11 was then refined by energy minimization using the program CNS [41]. In **Figure 5B**, an overlay of the F_v_ region of the parental αD11 and the model of the F_v_ region of the hum-αD11 is shown. The resulting model of the Fab fragment of hum-αD11 and the modeled F_v_ region being assembled in a composite immunoglobulin are shown in **Figure 5C**.

### Hum-αD11 shows an enhanced *in vitro* NGF binding affinity

In order to obtain the hum-αD11 as an antibody protein in the IgG1 format, for its functional evaluation and characterization, DNA sequences encoding hum-αD11 V_H_ and V_κ_ were synthesized using overlapping oligonucleotides (**Table S1**), genetically fused to the human γ1 heavy and the κ light constant regions, respectively (to reconstitute a human IgG1 antibody) and cloned in suitable eukaryotic expression vectors [42] that were used to co-transfect Chinese Hamster Ovary (CHO) mammalian cells.

**Figure 6:**
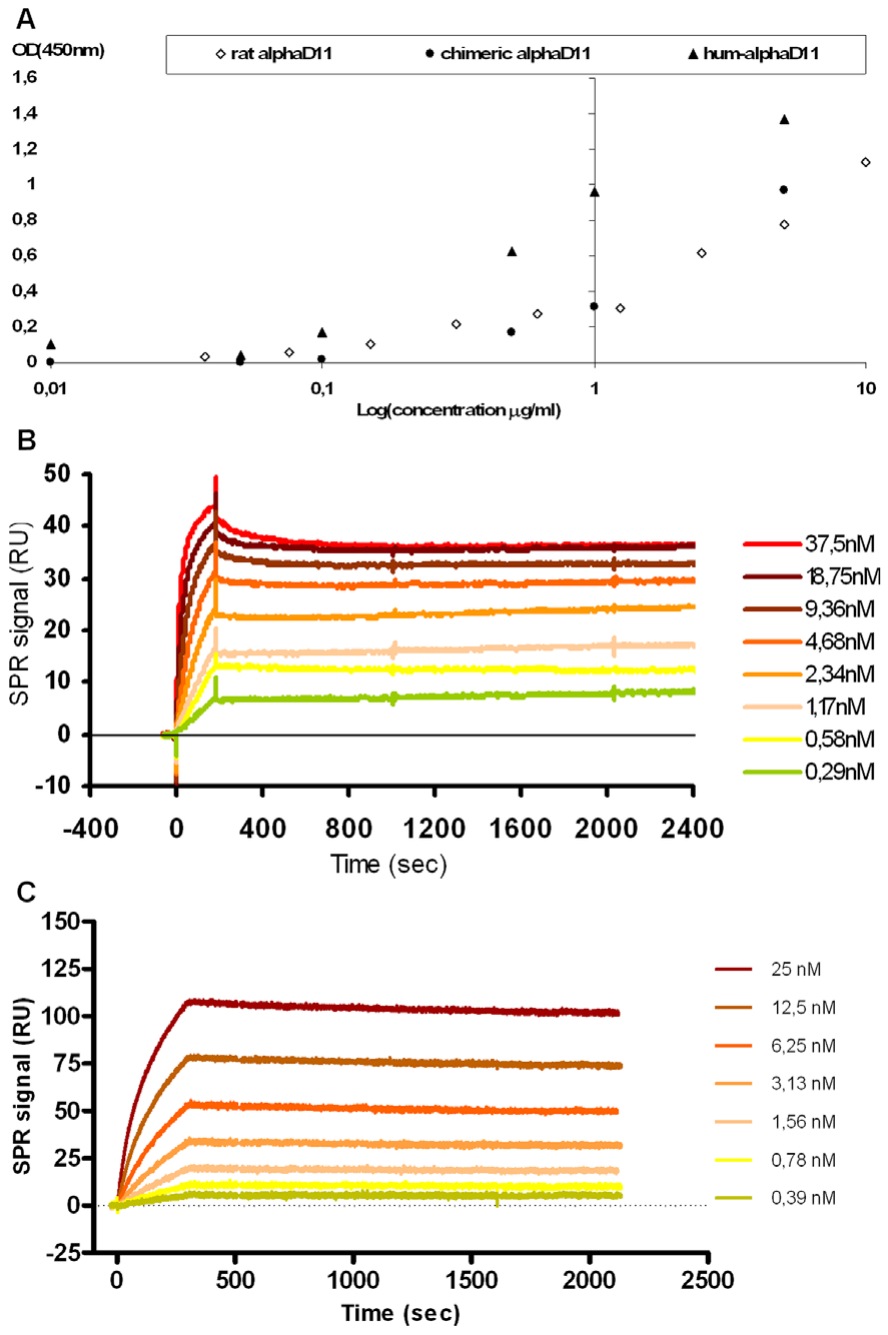
Hum-αD11 preserves the *in vitro* binding properties of the parental mAb αD11. (a) ELISA assay with mNGF coating (5 µg/ml); serial dilutions of parental mAb αD11, chimeric IgG1 αD11 and IgG1 hum-αD11, Protein A-Sepharose purified from transiently cotransfected CHO cells supernatants. (b) Binding curves of a range of concentrations (0.29–37.5 nM) of hNGF to immobilized parental mAb αD11 (immobilization level 1280.0 RU). (c) Binding curves of a range of concentrations (0.39–25.0 nM) of Fab hum-αD11 to immobilized hNGF (immobilization level 100.3 RU).

ELISA assay was performed on CHO transfectant supernatants, to evaluate the binding of IgG1 hum-αD11 binding to mNGF and compare it to the parental mAb αD11, expressed in the chimeric immunoglobulin IgG1 format, IgG1 chim- αD11 (rat αD11 variable regions fused to the γ1 heavy and the κ light constant regions, respectively) [22]. The IgG1 chim-αD11 and the rat mAb αD11 were previously shown to have overlapping NGF binding curves [22]. The chim-αD11 and hum-αD11 in the human IgG1 format were transiently expressed in CHO cells, purified by Protein A-Sepharose and quantified by immunoblot. After normalization, serial dilutions were tested by ELISA (**Figure 6A**). The results show that IgG1 hum-αD11 binds mNGF equally well as the IgG1 chim-αD11 (and the parental rat mAb αD11). Quite surprisingly, not only the NGF binding of the hum-αD11 IgG1 was not reduced, but on the contrary the binding affinity appeared to increase, in comparison to the chimeric IgG1 αD11 and to the parental rat mAb αD11 (**Figure 6A**). This might be the result of a more favorable interaction at the variable/constant domains interface in the two antibodies. Thus, the single-cycle humanization procedure was sufficient to fully reconstitute the NGF binding strength of the parental antibody, even showing some binding improvement.

To confirm the likely affinity improvement of the IgG1 hum-αD11 by kinetic and quantitative affinity measurements, Surface Plasmon Resonance (SPR) studies were performed, to compare the NGF binding kinetics with respect to the parental and humanized antibodies, by exploiting two different configurations aimed to avoid any avidity effect due to the use of the IgG1 format as an analyte. Details of the parental and humanized αD11 antibodies binding curves to hNGF are reported in **Figure 6B** and **Figure 6C**, respectively. The humanized version of the antibody, indeed showed a significantly higher affinity for hNGF with respect to the parental version, K_D_ values of 28.9±4.1 pM and 451±45 pM, respectively (**Table 1**).

**Table 1:** SPR analysis.

	k_a_ (1/Ms)	k_d_ (1/s)	K_D_ (pM)	K_A_ (1/M)
rat αD11	2.21×10^5^±0.22×10^5^	9.98×10^−5^±0.07×10^−5^	451±45	2.21×10^9^±0.22×10^9^
hum-αD11	3.80×10^5^±0.50×10^5^	1.10×10^−5^±0.06×10^−5^	28.9±4.1	3.40×10^10^±0.50×10^10^

Summary of the derived kinetic and equilibrium binding constants of parental IgG αD11 and Fab hum-αD11 towards hNGF.

### Hum-αD11 fully preserves the *in vitro* NGF antagonistic activity and the *in vivo* analgesic properties of the parental mAb αD11

To verify that the IgG1 hum-αD11 maintained the ability of the parental one to inhibit NGF biological activity *in vitro*, two cellular models were employed.

**Figure 7:**
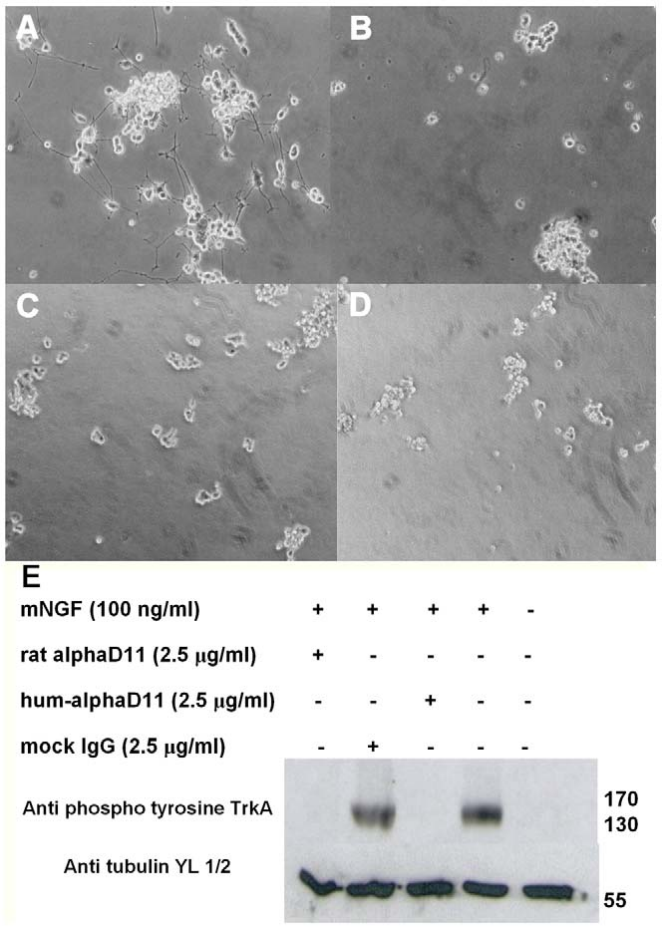
Hum-αD11 retains the biological activity of the parental mAb αD11 *in vitro*. (a–d) mNGF induced differentiation of PC-12 cells. Neurite outgrowth inhibition assay: photomicrographs of PC-12 cells, treated with (a) mNGF 100 ng/ml alone or preincubated (b) with mAb αD11 (5 µg/ml) or (c) with IgG1 hum-αD11 (5 µg/ml), concentrated from stable cotransfected CHO cells supernatants. (d) Negative control: untreated PC-12 cells. (e) TrkA phosphorylation inhibition assay in 3T3 TrkA cells. 3T3 TrkA cells were incubated with the indicated combinations of mNGF, parental mAb αD11, IgG1 hum-αD11 (purified by Protein A-Sepharose) and the irrelevant mAb SV5 as a negative control. Cell lysates were separated on a 10% SDS gel and phosphorylated TrkA detected using anti-phospho-Y490 TrkA Ab. The ubiquitous band of tubulin served as gel loading control.

First, in an NGF-induced neurite outgrowth bioassay on rat PC-12 cells [43] (**Figure 7A** to **Figure 7D**) both the parental mAb and the IgG1 hum-αD11 exerted identical effects. Indeed, mNGF treated PC-12 cells (**Figure 7A**), preincubated with either the parental mAb or the IgG1 hum-αD11, failed to show any neurite outgrowth (**Figure 7B** and **Figure 7C**), as in the absence of mNGF (**Figure 7D**). In control experiments NGF-induced differentiation occurs normally (**Figure 7A**), even if mNGF was preincubated with a non relevant mAb or with the concentrated untransfected CHO cell supernatants (data not shown). The nuclear morphology of IgG1 hum-αD11 treated PC-12, stained with 4′,6-diamidino-2-phenylindole (DAPI), under the different conditions of the assays, was normal (data not shown), underlining that the failure of cells treated with mNGF and IgG1 hum-αD11 to differentiate was not due to a non-specific toxic effect, but indeed to the neutralization of NGF binding and of the ensuing differentiation.

The ability of the parental mAb αD11 and the IgG1 hum-αD11 to inhibit the activation of human TrkA receptor by mNGF was compared in 3T3 TrkA cells (ectopically expressing human TrkA receptor, but not expressing the neurotrophin p75^NTR^ receptor). As shown by Western-blot analysis of TrkA phosphorylation at residue Y490 (**Figure 7E**), no activation of human TrkA receptor could be detected in the cells treated with mNGF preincubated either with the parental mAb αD11 or the IgG1 hum-αD11, as well as in the absence of mNGF. In the control experiments, mNGF-induced human TrkA activation occurred normally, also in the presence of a non relevant antibody.

At the functional level, the potency of the mAb hum-αD11 to neutralize the activity of NGF from different species was also ascertained by the TF-1 cell-based proliferation bioassay [33]. The mAb hum-αD11 inhibits TF-1 cell proliferation with a similar potency (IC_50_ value of *ca*. 30 ng/ml) in the presence of human, rat and mouse NGF, respectively (data not shown).

**Figure 8:**
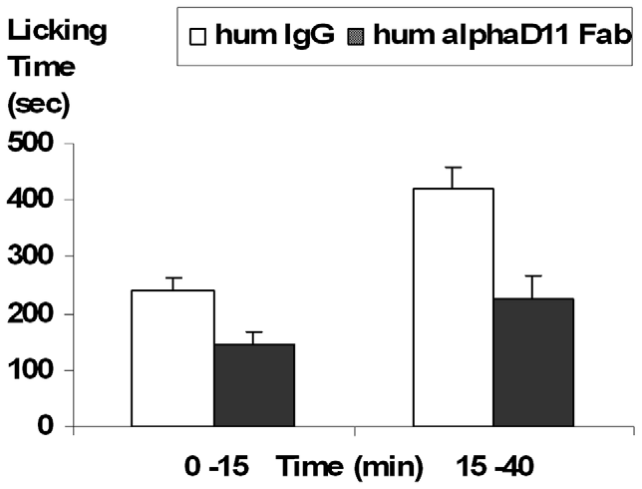
Analgesic activity of the hum-αD11 in the formalin-induced inflammatory pain model. Dose/Response effect of Fab hum-αD11 on the early (0–15 min) and late phase (15–40 min) in the course of the formalin test in CD1 mice. Treatment consisted in antibody injection (Fab hum-αD11 *vs* mock IgG) performed (in the same paw as for formalin) 45 min, before formalin injection and testing. Statistical analysis was performed on each phase (ANOVA and Fisher's Test for comparison of each couple of groups).

Finally the analgesic activity of the hum-αD11 antibody was tested in the formalin-induced inflammatory pain model *in vivo*. Since previous experiments with the parental mAb αD11 showed that analgesic effect was greater for mAb αD11 in the Fab than in the whole IgG format (**Figure 2B**), these experiments were performed with the Fab fragment of hum-αD11, expressed in the periplasm of bacterial cells. As shown in **Figure 8**, Fab hum-αD11, was able to reduce formalin-evoked pain both in the early and in the late phase (p<0.05) of the formalin test, with a stronger effect in the latter phase (which is related the inflammatory component of pain). The Fab hum-αD11 determined an identical analgesic response (halving of the pain response), at the same doses, as the parental rat-αD11 Fab fragment, and therefore it clearly retains the analgesic properties of the parental antibody.

## Discussion

In this paper we describe the humanization of rat mAb αD11, which potently antagonizes the activity of NGF, a target of great clinical relevance for various pathological situations, including presently untreatable forms of chronic and inflammatory pain [8], [15]. The humanized form of αD11 recapitulates the remarkable affinity (which is even improved) and neutralizing potency properties of the parental mAb αD11, including its analgesic properties in rodent models of inflammatory pain. The results obtained unequivocally prospect hum-αD11 as a lead therapeutic candidate for human pathologies where antagonizing systemic or local NGF activity in the periphery would be of clinical benefit.

The structure-based humanization strategy of mAb αD11 was performed by a novel method, which overcomes the well known limitations of the classical CDR grafting methods [44], [45], [46], whereby the CDRs of the parental rodent mAb are grafted onto human acceptor FWRs. The standard humanization procedure requires a laborious and time-consuming iterative procedure, to derive a humanized antibody with affinity and binding properties comparable to those of the parental one [47], [48], [49], [50]. Thus, the choice of the acceptor FWRs represents the critical point in the procedure of antibody humanization by CDRs grafting, leading most often to a significant binding affinity loss of the humanized antibody [51], [52], [53], due to distortions of the CDRs conformations by the human acceptor FWRs. This requires a trial and error iterative procedure, to correct structurally distorting residues and to reconstitute the original binding properties of the parental antibody. A universal combinatorial library of antibody FWRs suitable for humanizing exogenous antibodies by CDR-grafting, based on a bioinformatics approach, has been recently proposed [54] to address these issues. Even if computer modeling simulations and predictions can be employed to improve the outcome of humanization [44], [46], [55], the fidelity of antibody models to the experimental structures is rather low, especially concerning the CDR H3 loops, which are more variable in sequence, length and structure [56], [57], [58], [59] and more flexible [58], [60], [61] than the other CDRs. Moreover, the conformations of CDR H3 strongly depend on the neighbouring structural environment [58], and in a significant number of F_v_ crystal structures they are not even structurally defined. Therefore, reliable modelling of the CDR H3 is still a real challenge [62]. Moreover the overall conformation of antigen binding site of antibodies further depends in a complex and unpredictable manner on movements at the V_κ_/V_H_ interface [63]. Thus, antibody humanization by CDRs grafting, as such, can, and has proved to be, an unpredictably daunting and laborious task. On the other hand, the alternative method of F_v_ humanization by resurfacing, in which only solvent accessible residues of the non-human donor antibody are considered for substitution by homologous residues belonging to human FWRs regions [64], [65], [66], results in the presence of a higher number of non-human residues that might represent cryptic epitopes contributing to an immunogenic response in patients.

To overcome these drawbacks, we developed a new structure-based humanization strategy, to improve over traditional CDR grafting method. In the approach described here, we exploited the high resolution crystallographic structure of the Fab αD11 (PDB_ID: 1ZAN) [26], [27] in order to optimize the key step in the selection and choice of the human FWRs acceptors. This structure-based methodology allowed us to readily humanize the αD11 antibody in a single cycle, obtaining an engineered version whose binding and biological activity both *in vitro* and *in vivo* closely recapitulate those of the parental version. It is worth noting that the NGF binding affinity of the humanized hum-αD11 is not only maintained, but surprisingly and unpredictably improved, by an order of magnitude, over that of the parental antibody. The molecular underpinnings for such an unexpected affinity improvement will require a further structural investigation of the humanized antibody itself. With many antibodies of therapeutic interest being derived from murine monoclonal antibodies, the need for their humanization for use in patients is mandatory. The single-cycle structure-based method is a considerable improvement over the standard humanization methods, which are intrinsically empirical and require several trial and error refinement cycles. The method described here may significantly accelerate the clinical development path of many monoclonal antibodies of therapeutical interest.

The second major conclusion of this study establishes the anti-NGF hum-αD11 as an effective lead candidate for clinical applications in a therapeutic area that represents a severe unmet medical need. Pain is the most common symptom for which patients seek medical assistance, and most forms of chronic and inflammatory pain are inadequately treated by present therapeutic options. Compelling evidence has accumulated, demonstrating that the NGF-TrkA system is a key modulator of inflammatory and nociceptive responses and a master control system for pain, functionally placed upstream in the hierarchy of the pain regulation process [6], [67]. There is, therefore, a strong rationale for the development of a new generation of analgesic therapeutic interventions, based on antagonising the NGF pathway via inhibition of its interactions to receptors [8], [15]. In this respect the large and rather flat ligand-receptor binding interfaces disclosed by the crystal structures of the complexes between NGF and its receptors p75^NTR^ and TrkA [29], [68], [69], make the rational design of high affinity and specific small-molecule antagonists of these interacting surfaces a daunting prospect. Moreover, small molecule NGF-TrkA antagonists are more likely than larger antibody molecules to cross the blood-brain barrier, leading to unwanted neurological side effects. For these reasons the use of function-blocking antibodies targeting the NGF ligand [27], [70] or its TrkA receptor [71], [72], [73] represent one obvious strategy to develop new pain therapeutics [8], [15].

The first antibody in this group reaching clinical evaluation in humans is tanezumab, a humanized anti-NGF monoclonal antibody [70], [74]. The therapeutic concept of blocking the activity of NGF for chronic and inflammatory pain received a very strong validation from the results of proof-of concept clinical trials showing that tanezumab can very effectively and persistently relieve joint pain and improve functions in moderate-to-severe osteoarthritis patients [75]. However, in a subsequent Phase 3 clinical study of tanezumab for osteoarthritis of the hip and knee, 16 treated subjects showed joint failure and required total joint replacement [16], [75]. This led the U.S Food and Drug Administration to put on hold the clinical programs for tanezumab, until more information on the incidence and causes of these adverse events is gained (http://clinicaltrials.gov/ct2/results?term=ngfantibody). While these events could be ascribed to causes to be determined, related to the NGF/TrkA system *in vivo*, antibody-specific causes cannot be excluded and should be considered. In this respect, comparative evaluations of different anti-NGF antibodies will be very informative.

Although several mAbs were raised against NGF (reviewed in [74]), the mAb αD11 deserves a special interest, not only because of its picomolar affinity [19], antagonistic potency and specificity, but also because its NGF neutralizing properties have been very extensively verified in a diverse set of *in vivo* situations [20], [21], [22], [23], [24], [25] and because it is the only anti-NGF monoclonal antibody for which a 3D structure has been derived [26], [27]. We now further show that the anti-NGF mAb αD11 displays very significant NGF antagonistic properties in relevant murine pain models, with a remarkable and surprising long-lasting analgesic property that may be of high clinical relevance. Although the mechanistic dissection of this long-term analgesic effect remain to be further characterized, it appears to be directly related to NGF-TrkA signaling in adult nociceptor neurons, since a similar long-lasting persistent analgesic effect was observed after administration of the neutralizing anti-TrkA MNAC13 antibody [73]. Thus, interrupting the activation of NGF-TrkA signaling in chronic and inflammatory pain states might induce a therapeutically beneficial feedback loop, prospecting anti-NGF ad anti-TrkA antibodies as analgesics with disease-modifying properties.

These results establish hum-αD11, the humanized counterpart of mAb αD11, as a lead candidate with a strong therapeutic potential not only in the pain arena, but for all those pathological states where an excess of NGF expression and/or activity is detrimental.

## Materials and Methods

### Construction and cloning of the cDNAs of the variable regions of hum-αD11

cDNAs of hum-αD11 V_H_ and V_κ_ were obtained by gene synthesis using overlapping oligonucleotides (**Table S1**) according to Kolbinger *et al.* [76]. After overlap assembly PCR, fragments of the correct size were purified from agarose gel and directionally cloned in expression vectors for IgG1 expression [42], respectively *BssHII/BstEII* in V_H_ Express vector for hum-αD11 V_H_ and *ApaLI/BglII* in V_κ_ Express vector for hum-αD11V_κ_.

In order to produce Fab hum-αD11 in the bacterial periplasm, the following two expression cassettes were cloned to express hum-αD11 V_H_ and hum-αD11 V_κ_ in fusion with human constant regions of the heavy (hC_H_) and the light (hC_κ_) chains. In details, hum-αD11 V_κ_ was directionally cloned (*NcoI/HindIII*) in frame with hC_κ_ (*HindIII/NotI*) in pET22b (Novagen) to obtain hum-αD11 light chain in pET22b. After inserting the PelB secretion sequence (*NdeI/NcoI*) in pET28 (Novagen), hum-αD11 V_H_ was directionally cloned (*NcoI/NheI*) in frame with hC_H_ (*NheI/XhoI*) and with C-terminal his-tag to obtain hum-αD11 heavy chain in pET28_ PelB.

At each cloning step, positive clones, isolated by PCR screening directly on bacterial colonies, were confirmed by DNA sequencing.

### Expression of hum-αD11 in the IgG1 and Fab formats

The CHO cells (The European Collection of Cell Cultures, Sigma-Aldrich, Product n° 85050302), grown in 90mm dishes, were transfected with 3 µg IgG1 hum-αD11 expression plasmids (1.5 µg of hum-αD11 V_H_ in V_H_ Express and 1.5 µg of hum-αD11 V_κ_ in V_κ_ Express) and with 3 µg chimeric αD11 expression plasmids (1.5 µg of rat αD11V_H_ in V_H_ Express and 1.5 µg of rat αD11 V_κ_ in V_κ_ Express) [22], using FuGENE (Roche) according to the manufacturer's protocols. Conditioned medium was collected 72 hrs after transfection.

To select double transfectant clones expressing IgG1 hum-αD11, CHO cells were cotransfected as reported above, using the same amount of *EcoRI* linearized plasmids. 36 hrs after transfection, micophenolic acid (50 µg/ml) and G418 (500 µg/ml) were added. Conditioned media were concentrated to 0.2 ml final volume using Microcon concentrators (Millipore) or purified using Protein A-Sepharose (GE Healthcare).

Protein concentrations were estimated by immunoblot. In details 5 µl of each purified sample or concentrated conditioned medium were spotted on a nitrocellulose membrane. After blocking with PBS (5% non fat dry milk), the membrane was incubated at first with an anti-human polyclonal antibody (Pierce) diluted 1∶500 in PBS (5% non fat dry milk), acting as primary Ab, followed by the anti-goat Ab, coupled to horseradish peroxidase (Dako), diluted 1∶1000. The Horseradish Peroxidase (HRP) conjugates were detected with the electrochemiluminescence protocol developed by Amersham Corp.

The amounts of human IgG in the different samples were normalized after densitometric scanning and standards of purified human IgG (Sigma) were used to determine absolute protein levels; as negative control for concentrated conditioned media, concentrated CHO supernatant of untransfected cells was used.

In order to express hum-αD11 Fab, hum-αD11 heavy chain in pET28_PelB and hum-αD11 light chain in pET22b were co-transformed in BL21(DE3) E.coli cells. Cells were grown at 30°C in M9CA broth supplied with kanamycin (50 µg/ml) and ampicillin (100 µg/ml), induced at an OD_600 nm_ of 0.9 by adding 0.5 mM IPTG and left to grow for 15 hrs. The cell pellet resuspended in buffer A (50 mM Na phosphate pH 8, 0.5 M NaCl, 5 mM MgCl_2,_ 10 mM imidazole, 5% glycerol) was incubated with 1 mM PMSF, 100 µg/ml lysozyme and 5 µg/ml Dnase I, for 30 min and then sonicated on ice. Soluble extract was loaded on Ni-NTA Superflow resin (Qiagen) equilibrated with buffer A and Fab hum-αD11 was subsequently eluted with 250 mM imidazole. The collected fractions were then pooled and loaded on a HiLoad 16/60 Superdex 75 (GE Healthcare) pre-equilibrated with 20 mM Tris-HCl pH 8.5, 0.2 M NaCl, 5 mM MgCl_2,_ 5% glycerol. The fractions corresponding to the Fab hum-αD11 were concentrated using an Amicon Ultra-15 centrifugal filter unit (Millipore) with a membrane cut-off of 10 kDa.

### ELISA assays

ELISA assays were performed according to Covaceuszach *et al.* [72] with the following modifications.

In order to compare mAb αD11 binding towards human and mouse NGF, mNGF (Alomone) and hNGF obtained according to Covaceuszach *et al.* [77], were coated; followed by first serial dilutions of mAb αD11 (1∶2 dilutions in the concentration range between 20 µg/ml and 18 ng/ml), acting as primary antibody, and finally by the anti-rat antibody peroxidase conjugated (Dako) acting as secondary antibody.

ELISA assay to compare chimeric and IgG1 hum-αD11 binding towards mNGF (Alomone) was performed with the following modifications: serial dilutions of chimeric and IgG1 hum-αD11 were incubated and the anti-human polyclonal antibody (Pierce) was used as primary antibody and the anti-goat antibody peroxidase conjugated as secondary antibody (Dako). All the experiments were done in duplicate.

### Surface Plasmon Resonance

SPR measurements were performed with a BIACore instrument (BIACore AB, Uppsala, Sweden) in triplicate.

Parental rat mAb αD11 was immobilized on three CM5 sensor chips by cross-linking the amine groups according to the manufacturer's instructions, obtaining SPR signals, after completion of the chip regeneration cycles, respectively of 1025, 1040 and 1280 resonance units (RU). The binding kinetics for rat mAb αD11 were determined by injection on each surface of serial dilutions (in the 0.29 nM to 37.5 nM concentration range) of hNGF in PBS buffer with addition of 0.005% v/v Surfactant P20 at a flow rate of 30 µl/min.

The binding kinetics for Fab hum-αD11 were determined by immobilizing hNGF on three CM5 sensor chips by cross-linking the amine groups according to the manufacturer's instructions, obtaining SPR signals, after completion of the chip regeneration cycle, respectively of 89, 93 and 100 resonance units. Serial dilutions (in the 0.39 nM to 25 nM concentration range) of Fab hum-αD11 (kindly provided by PanGenetics UK Ltd.) in HBSEP (10 mM HEPES, 150 mM NaCl, 3 mM EDTA and 0.005% Surfactant P20, pH 7.4) containing 100 µg/ml bovine serum albumin, were injected on each surface at a flow rate of 50 µl/min.

Data were analyzed using the BIAevaluation 3.0 package (GE Healthcare) to yield the apparent equilibrium constant K_D_ (defined as the k_a_/k_d_ ratio) and K_A_ (defined as the k_d_/k_a_ ratio).

### NGF bioassay with PC-12 cells

Rat adrenal gland phaeochromocytoma PC-12 cells (Sigma-Aldrich, Product n° 88022401) [43] were maintained in RPMI 1640 medium (Life Technologies, Milano, Italy), supplemented with 10% fetal calf serum. PC-12 cells were primed with 50 ng/ml mNGF (Alomone) on collagen-coated (type I, BD Biosciences) 35 mm Petri dishes at a density of 0,25×10^5^ cells per dish for the second day after seeding onward. Priming was carried out for 5–6 days, with mNGF added every 3 days, then cells were detached and plated on collagen-coated 35 mm Petri dishes. For differentiation assays, cells were incubated with 100 ng/ml mNGF for 2–4 days in the presence or absence of parental mAb or IgG1 hum-αD11 (5 µg/ml; 1 hour preincubation).

### TrkA Phosphorylation-Inhibition Assay

TrkA Phosphorylation-Inhibition Assay was performed according to Ugolini *et al.* [73], with the following modification. Prior treatment, 100 ng/ml mNGF was preincubated in the presence or absence of parental mAb αD11, IgG1 hum-αD11 or irrelevant mAb SV5 (2.5 µg/ml) in serum-free medium supplemented with 0.05% BSA for 1 hour.

BALB/C 3T3-transfected cells (3T3-TrkA), mouse embryonic fibroblast cell line stable transfected with human TrkA full-length, were kindly provided by Dr. Stefano Alemà [Consiglio Nazionale delle Ricerche, Istituto di Biologia Cellulare e Neurobiologia, Via E. Ramarini 32, I-00015 Monterotondo Scalo (Roma), Italy].

The human premyeloid cell line TF-1 was purchased from the American Type Culture Collection (ATCC, Product n° CRL-2003).

### Inflammatory and neuropathic pain models

Formalin test and neuropathic pain tests were performed according to Ugolini *et al.* [73].

In particular, regarding the neuropathic pain model, the CCI of the sciatic nerve [34] was performed following two treatment protocols, *i.e.* Protocol A (short lasting): 4 I.P. administrations at days 3, 4, 5, 6 after surgery of mAb αD11 (single dose: 50 µg/injection, roughly equivalent to 1.4 mg/Kg) and Protocol B (long lasting): 8 I.P. administrations at days 3, 4, 5, 6, 7, 8, 9, 10 after surgery of mAb αD11 (single dose: 70 µg/injection, roughly equivalent to 2 mg/Kg).

### Ethics Statement

All animal work has been approved by Italian Ministry of Health (order N.34/2008-B) and have been conducted according to the Italian National law (DL116/92, Application of the European Communities Council Directive 86/609/EEC) on care and handling of the animals and with the guidelines of the Committee for Research and Ethical Issues of International Association for the Study of Pain [78].

## Supporting Information

Figure S1 **Primary and tertiary structural comparison.** 3D structural and sequence comparisons between the crystal structure of the Fab rat αD11 and the crystal structures of human or humanized antibodies, Fabs, IgGs or of their complexes with antigens indentified in the PDB database (release #101, July 2002). The plotted variables are: The skeleton C^α^ r.m.s.d , the % of C^α^ atoms considered in the r.m.s.d calculations and A) % of sequence homology on both the variable domains B) % of sequence identity on both the variable domains C) % of sequence homology on the FWRs D) % of sequence identity on the FWRs.
(TIF)

Table S1 **Oligonucleotides sequences used in the synthesis of the CDRs grafted hum-αD11 V_k_ (A) and V_H_ (B) regions by overlap-assembly PCR.**
(DOC)
