# Supplementary figures and images for: Single Cycle Structure-Based Humanization of an Anti-Nerve Growth Factor Therapeutic Antibody
Source: PLoS One. 2012 Mar 5;7(3):e32212. doi: 10.1371/journal.pone.0032212 (PMC3293900; doi:10.1371/journal.pone.0032212)

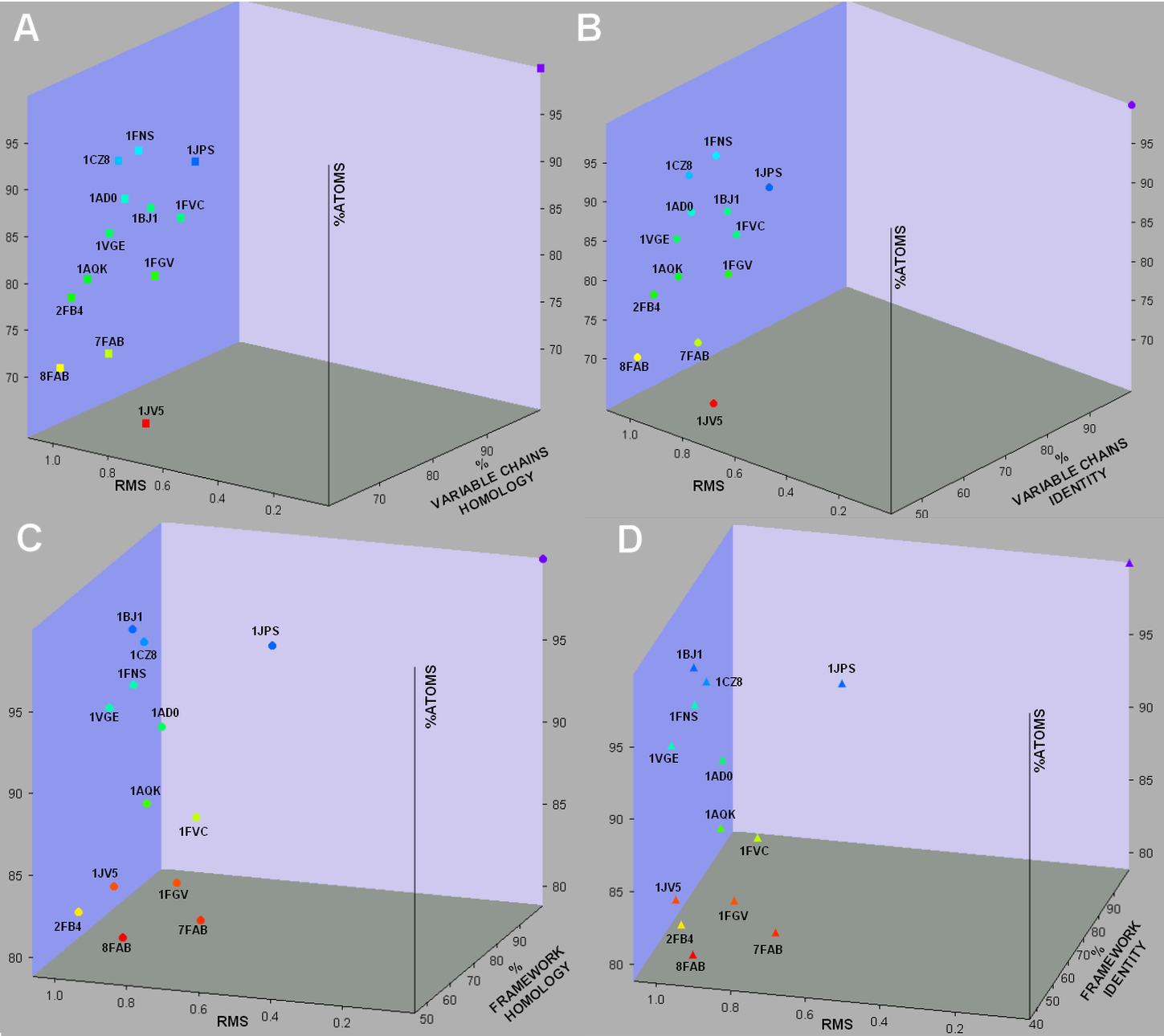

Supplement: Figure S1 — Primary and tertiary structural comparison. 3D structural and sequence comparisons between the crystal structure of the Fab rat αD11 and the crystal structures of human or humanized antibodies, Fabs, IgGs or of their complexes with antigens indentified in the PDB database (release #101, July 2002). The plotted variables are: The skeleton Cα r.m.s.d , the % of Cα atoms considered in the r.m.s.d calculations and A) % of sequence homology on both the variable domains B) % of sequence identity on both the variable domains C) % of sequence homology on the FWRs D) % of sequence identity on the FWRs. (TIF) [file pone.0032212.s001.tif]
