# Supplementary material for: Single Cycle Structure-Based Humanization of an Anti-Nerve Growth Factor Therapeutic Antibody
Source: PLoS One. 2012 Mar 5;7(3):e32212. doi: 10.1371/journal.pone.0032212 (PMC3293900; doi:10.1371/journal.pone.0032212)
Supplement: Table S1 — Oligonucleotides sequences used in the synthesis of the CDRs grafted hum-αD11 Vk (A) and VH (B) regions by overlap-assembly PCR. (DOC) [file pone.0032212.s002.doc]

**A.**

OligoL1S:

5’ACAGGCGTGCACTCCGACATCCAGACCCAGTCTCCATCTTCCCTGTCTGCATCTGTGGGAGACCGCGTCACCATC 3’

OligoL2AS: 5’TGGCTTCTGCTGATACCATGCTAAAGCATTATAAATGTCCTCACTTGCTCGACATGTGATGGTGACGCGGTCTCCCAC 3’

OligoL3S: 5’GCATGGTATCAGCAGAAGCCAGGGAAAGCTCCTAAGCTCCTGATCTATAATACAGATACCTTGCATACAGGGGTCCCA 3’

OligoL4AS: 5’CAGGCTGCTTATCGTGAGAGTATAGTCTGTACCAGATCCACTGCCACTGAATCGTGATGGGACCCCTGTATGCAAGGT 3’

OligoL5S:

5’ACTCTCACGATAAGCAGCCTGCAACCTGAAGATTTCGCAACTTATTTCTGTCAGCACTATTTCCATTATCCTCGG 3’

OligoL6AS:

5’CAATCTAGAATTCTACTCACGTTTGATCTCCACCTTGGTCCCTTGACCGAACGTCCGAGGATAATGGAAATAGTG 3’

External VkS:

5’ACAGGCGTGCACTCCGAC 3’

External VkAS:

5’CAATCTAGAATTCTACTCACG 3’

**B.**

OligoH1S:

5’ACAGGCGCGCACTCCGAGGTGCAGCTGGTGGAATCAGGAGGTGGTCTGGTGCAGCCCGGAGGGTCCCTGCGCCTCAGCTGC 3’

OligoH2AS: 5’TCCTGGAGCCTGTCGAACCCAGTTCACATTGTTGTTGGTTAGTGAGAAGCCAGAGGCAGCGCAGCTGAGGCGCAGGGACCC 3’

OligoH3S: 5’AACTGGGTTCGACAGGCTCCAGGAAAAGGTCTGGAGTGGGTGGGAGGAGTCTGGGCTGGTGGAGCCACAGATTACAATTCA 3’

OligoH4AS: 5’CATTTGTAAGTAAGCTGTGTTCTTGGAGTTGTCGCGACTGATGGTGAATCGGGATTTGAGAGCTGAATTGTAATCTGTGGCTCC 3’

OligoH5S: 5’AAGAACACAGCTTACTTACAAATGAACAGTCTGCGCGCTGAAGACACAGCCGTTTATACTGTGCCAGAGACGGGGGCTATAGC 3’

OligoH6AS: 5’TGAGGAGACGGTGACCAGAGTTCCTTGACCCCAGGCATCCATAGCATAGAGGGTAGAGCTGCTATAGCCCCCGTCTCTGGC 3’

External VHS:

5’ ACAGGCGCGCACTCCGA 3’

External VHAS:

5’ TGAGGAGACGGTGACCAG 3’
